# The role of carbon starvation in the induction of enzymes that degrade plant-derived carbohydrates in *Aspergillus niger*

**DOI:** 10.1016/j.fgb.2014.04.006

**Published:** 2014-11

**Authors:** Jolanda M. van Munster, Paul Daly, Stéphane Delmas, Steven T. Pullan, Martin J. Blythe, Sunir Malla, Matthew Kokolski, Emelie C.M. Noltorp, Kristin Wennberg, Richard Fetherston, Richard Beniston, Xiaolan Yu, Paul Dupree, David B. Archer

**Affiliations:** a School of Life Sciences, University of Nottingham, University Park, Nottingham NG7 2RD, UK; b Deep Seq, Faculty of Medicine and Health Sciences, Queen’s Medical Centre, University of Nottingham, Nottingham NG7 2UH, UK; c Biological Mass Spectrometry Facility biOMICS, University of Sheffield, Brook Hill Road, Sheffield S3 7HF, UK; d Department of Biochemistry, University of Cambridge, Tennis Court Road, Cambridge CB2 1QW, UK

**Keywords:** pNP-Cel, 4-Nitrophenyl-β-d-cellobioside, pNP-Ara, 4-Nitrophenyl-α-l-arabinofuranoside, pNP-β-Glc, 4-Nitrophenyl-β-d-glucopyranoside, pNP-Xyl, 4-Nitrophenyl-β-d-xylopyranoside, pNP-α-Glc, 4-Nitrophenyl-α-d-glucopyranoside, MWCO, molecular weight cut-off, DASH, DNA sequencer-assisted saccharide analysis in high throughput, DP, degree of polymerization, *Aspergillus niger*, Lignocellulose, CAZy enzymes, Transcriptome, Carbon starvation, Inducer

## Abstract

Fungi are an important source of enzymes for saccharification of plant polysaccharides and production of biofuels. Understanding of the regulation and induction of expression of genes encoding these enzymes is still incomplete. To explore the induction mechanism, we analysed the response of the industrially important fungus *Aspergillus niger* to wheat straw, with a focus on events occurring shortly after exposure to the substrate. RNA sequencing showed that the transcriptional response after 6 h of exposure to wheat straw was very different from the response at 24 h of exposure to the same substrate. For example, less than half of the genes encoding carbohydrate active enzymes that were induced after 24 h of exposure to wheat straw, were also induced after 6 h exposure. Importantly, over a third of the genes induced after 6 h of exposure to wheat straw were also induced during 6 h of carbon starvation, indicating that carbon starvation is probably an important factor in the early response to wheat straw. The up-regulation of the expression of a high number of genes encoding CAZymes that are active on plant-derived carbohydrates during early carbon starvation suggests that these enzymes could be involved in a scouting role during starvation, releasing inducing sugars from complex plant polysaccharides. We show, using proteomics, that carbon-starved cultures indeed release CAZymes with predicted activity on plant polysaccharides. Analysis of the enzymatic activity and the reaction products, indicates that these proteins are enzymes that can degrade various plant polysaccharides to generate both known, as well as potentially new, inducers of CAZymes.

## Highlights

• The transriptomic response of *Aspergillus niger* to wheat straw is sequential.
• The early response consists of genes encoding hemicellulolytic enzymes.
• The later response to straw consists of genes encoding cellulases and pectinases.
• The early response to carbon starvation overlaps with that to wheat straw.
• CAZymes in starved cultures release mono- and oligosaccharides from lignocellulose.

## 1 Introduction

Wheat straw is the most abundant possible feed stock for biofuel production from agricultural wastes in Europe (Talebnia et al., 2010). Enzymatic saccharification of plant waste material, such as wheat straw, transforms complex carbohydrates in the lignocellulose of the plant cell wall into simple sugars. These sugars can then be converted to ethanol or other biofuels. One of the bottlenecks in this enzymatic saccharification is the cost of the enzymes. More efficient production of enzymes, as well as the improvement of their activity could help to improve the production of second generation biofuels (Chundawat et al., 2011).

The plant cell wall consists mainly of cellulose, hemicellulose, pectin and lignin. While cellulose is a linear molecule consisting of β-1,4-linked glucose, hemicelluloses vary in relative abundance and composition. They share a β-1,4 backbone structure which can consist of glucose, mannose or xylose residues. The most abundant hemicellulose in wheat straw is arabinoxylan, a xylan that can be decorated with, for example, α-arabinofuranose, glucuronic acid residues, and acetyl groups. Other hemicelluloses include xyloglucan and galactoglucomannan (Scheller and Ulvskov, 2010). Pectin makes up a small part of the cell wall in grasses such as wheat straw but is abundant in fruits, and plants such as *Arabidopsis thaliana* (Harholt et al., 2010). Pectin polysaccharides have a backbone of α-1,4-linked galacturonic acid or alternating α-1,2/α-1,4 linked galacturonic acid and rhamnose. Backbone decorations can range from simple monomers to complex side chains such as arabinan and arabinogalactan (Caffall and Mohnen, 2009, Harholt et al., 2010).

To hydrolyse this complex structure of the plant cell wall efficiently, an enzyme cocktail is required that contains a variety of activities. Fungal saprobes such as *Aspergillus niger*, *Trichoderma reesei* or *Neurospora crassa* are well equipped to hydrolyse plant cell walls, and their genomes encode a large array of glycoside hydrolases, carbohydrate esterases and polysaccharide lyases, as well as accessory proteins (Borkovich et al., 2004, Martinez et al., 2008, Pel et al., 2007). Recent research has aimed at investigating the degradative response of such fungi towards lignocellulose (Andersen et al., 2012, Benz et al., 2014, de Souza et al., 2011, Hakkinen et al., 2012, Kubicek, 2013, Ries et al., 2013) and the regulatory mechanisms behind this response (Coradetti et al., 2013, de Souza et al., 2013), and the progress has been reviewed (Culleton et al., 2013, Glass et al., 2013, Znameroski and Glass, 2013). Understanding the responses of filamentous fungi to lignocellulose may result in new strategies to improve enzyme mixtures for biofuel production, by suggesting new enzyme activities, identifying potential accessory proteins which can assist enzyme function, and/or by exploring native gene induction systems to allow more efficient production of lytic enzymes in fungal hosts. The industrially important fungus *A. niger* is an excellent model organism for this because of its broad range of hydrolytic enzymes and availability of both a large body of background knowledge and a wide variety of tools (Andersen et al., 2012, Couturier et al., 2012).

When *A. niger* is exposed to a complex substrate such as lignocellulose, transcription of a large number of genes encoding carbohydrate active enzymes (CAZymes) is activated (Delmas et al., 2012, de Souza et al., 2011, de Souza et al., 2013) and proteins that are active on a broad range of lignocellulosic substrates are secreted (Couturier et al., 2012, de Souza et al., 2011). The transcriptional response of *A. niger* to wheat straw is sequential. For example, the genes encoding enzymes cellobiohydrolase CbhB and arabinofuranosidase AfbB were induced after 6 h of exposure to straw, while the genes encoding enzymes cellobiohydrolase CbhA and a putative lytic polysaccharide monooxygenase were expressed only after 9 h of exposure (Delmas et al., 2012). These genes are considered to be part of the early and major degradative response of *A. niger* respectively. The gene *creA* encodes a transcriptional regulator that mediates carbon catabolite repression in the presence of easily metabolised carbohydrates such as glucose, reviewed in (Ruijter and Visser, 1997). Relief of carbon catabolite repression, by glucose depletion or deletion of *creA*, was found to activate expression of genes of this early degradative response, while expression levels of genes of the major, later, degradative response were not affected (Delmas et al., 2012). The possible function of enzymes encoded by these and other genes that are responsive to carbon catabolite (de)repression, would be in the generation of soluble, low molecular weight carbohydrates that act as inducers for other genes encoding CAZymes, either working on their own or together with constitutively-expressed enzymes (Martens-Uzunova and Schaap, 2009). The observed sequential gene induction on wheat straw thus fits a model where carbon starvation allows low level expression of a set of CAZy enzymes, which can scout for available substrates and act on them to release inducer molecules that are needed for the full induction of an appropriate set of substrate specific hydrolytic enzymes (Delmas et al., 2012). Similarly, in *N. crassa* a number of genes that encode cellulolytic and pectinolytic enzymes show increased transcription levels upon carbon starvation as well as during early exposure to cellulose or pectin (Benz et al., 2014, Coradetti et al., 2012). Thus, similar conclusions can be drawn for the role of carbon starvation in the induction of CAZy enzymes in *N. crassa* during exposure to isolated cell wall polysaccharides (Znameroski et al., 2012, Znameroski and Glass, 2013). Also, deletion of *cre1* from *T. reesei* resulted in up-regulation of genes encoding cellulases and hemicellulases during growth on glucose (Nakari-Setala et al., 2009). The conservation of the response of genes encoding hemicellulases and cellulases to carbon catabolite de-repression in several ascomycetes, suggests conservation of the role of carbon starvation in the induction of lignocellulose-degrading CAZymes during fungal exposure to lignocellulose.

No systematic overview is available of early events during the induction of genes encoding plant polysaccharide-degrading enzymes during the exposure of fungi to complex lignocellulosic substrates. Here, we investigate the full scope of the early and major degradative response of *A. niger* to wheat straw, as well as the early and late carbon starvation response using RNA-Seq. We show that during both exposure to wheat straw and during carbon starvation, the early events (6 h) are significantly different compared to later events (24 h). Importantly, the response of *A. niger* during early exposure to straw largely overlaps with the early carbon starvation response, confirming the importance of carbon starvation during induction of CAZymes on complex lignocellulosic material. We show that filtrate from carbon-starved cultures indeed contains enzymatic activities that are capable of degrading plant-derived polysaccharides. These enzymes can generate mono- and oligosaccharides that could act as inducers of genes encoding CAZymes that are active on lignocellulose.

## 2 Materials and methods

### 2.1 Strains and growth conditions

*A. niger* strain N402 (Bos et al., 1988) or AB4.1 (Hartingsveldt et al., 1987) was routinely grown on potato dextrose agar (PDA, Oxoid) or aspergillus minimal medium (AMM) (Delmas et al., 2012). Medium contained 1% (w/v) glucose or 1% ball milled wheat straw (Delmas et al., 2012) as the sole carbon source. Fungal strains were grown on agar slopes at 28 °C to produce spores, which were harvested using 0.1% (v/v) Tween 20. For liquid cultures, 100 ml medium in a 250 ml flask was inoculated with 1 × 10^6^ spores ml^−1^ and incubated at 28 °C, 150 RPM. For cultivation on wheat straw or in medium without carbon source, AMM containing glucose was inoculated with spores and incubated for 48 h to generate mycelium. Mycelium was collected by filtration over Miracloth (Calbiochem), washed with AMM without carbon source and subsequently 1.5 g wet weight of mycelium was transferred to a flask with the appropriate medium.

### 2.2 RNA isolation, sequencing and data analysis

*A. niger* N402 was grown in duplicate cultures as described in Section 2.1 for 6 and 24 h in AMM without carbon source, as well as for 6 h on AMM with wheat straw. Mycelium was harvested from each of these time-points, as well as from the culture grown in AMM with glucose. After grinding the frozen mycelium to powder under liquid nitrogen using a pestle and mortar, RNA was isolated using the Plant/Fungi total RNA Purification Kit (Norgen Biotek, Canada) following the manufacturer’s instructions, including the on-column DNAse treatment step. The concentration and quality of RNA for each sample was determined by UV spectrometry (Nanodrop ND-1000 spectrophotometer).

A total of 10 μg of total RNA was depleted of ribosomal RNA using the Ribominus Eukaryotic kit (Invitrogen). Transcriptome libraries were prepared and sequenced as described (Delmas et al., 2012) with the following exceptions; the KAPA library quantification kit for Applied Biosystems SOLiD platform was used to quantify the libraries by qPCR. Emulsion PCR and bead-based enrichment was carried out using the SOLiD EZ bead system. Using a SOLiD 5500xl Life Technologies sequencer, 50 bp/35 bp colourspace paired-end reads were generated.

The Life Technologies LifeScope (v2.5.1) Whole Transcriptome (WT) Pipeline was used to filter, then map the SOLiD reads to the reference genome sequence described before (Delmas et al., 2012) using the mate-pair read WT pipeline. The RNA-Seq data obtained previously (Delmas et al., 2012) of *A. niger* exposed to wheat straw for 24 h, as well as the data from the corresponding glucose culture, were reanalysed using the same WT pipeline to allow data comparison. Reads from this dataset were mapped using the single fragment WT pipeline. For both data sets, reads were initially filtered against library adaptor and barcode sequences as well as *A. niger* rRNA 5.8S, 16S, 18S & 28S sequences obtained from Genbank. Reads that passed the filter were then mapped to the reference genome sequence, and to a library of exon junction sequences derived from the genome sequence using known exon coordinates. This allowed reads that spanned exon junctions (spliced reads) to be determined. Read counts per gene were determined using the program Htseq-count (http://www-huber.embl.de/users/anders/HTSeq) using uniquely aligned reads with ⩾MAPQ20. For paired read alignments only the forward read (F3) were counted. The count information was then used to calculate normalized gene expression values as RPKM (Mortazavi et al., 2008). Read counts were also used as the input for calculating differentially expressed (DE) genes using the R package DESeq (version 1.9) (Anders and Huber, 2010). An adjusted *p*-value of 0.05 was the significance threshold (*p*-value adjusted for multiple testing with the Benjamini–Hochberg procedure for false discovery rate (FDR)). Unless specified otherwise, differentially-expressed genes discussed in this paper met the threshold for significance and had a RPKM fold change of ⩾3.0. The RNA-Seq data obtained previously for 24 h exposure to wheat straw (Delmas et al., 2012) is available at the Gene Expression Omnibus (GEO) database (Barrett et al., 2013) under accession number GSE33852 and the data obtained for 6 h exposure to wheat straw as well as 6 h and 24 h of carbon starvation are available under accession number GSE57315. Supplementary Table S1 contains the expression levels for all genes.

**G**ene **O**ntology (GO) enrichment analysis was performed using FetGOat http://www.broadinstitute.org/fetgoat/ (Nitsche et al., 2011). The *A. niger* CBS 513.88 strain, a critical FDR q-value of 0.05, a minimal annotation group size of 2 and the Benjamini and Hochberg multiple testing correction were selected. All three ontologies were analysed for both over-represented and under-represented gene sets. Separate lists of genes annotated in CBS 513.88 genome with increased or decreased transcript abundance that had adjusted *p*-values ⩽0.05 (DESeq) and a fold change of ⩽or⩾3-fold compared to the respective 48 h glucose control were analysed. In the list of genes with increased transcript abundance, genes were also included which had a zero value in the control condition but were expressed in the test condition and had an adjusted *p*-value ⩽0.05. More detailed results of the enrichment analyses as well as the gene lists used for the enrichment analyses are included in Supplementary Table S2.

Clustering was performed using the GenePattern software platform http://www.broadinstitute.org/cancer/software/genepattern/ (Reich et al., 2006) where the Hierarchical Clustering module version 5 was used (de Hoon et al., 2004, Eisen et al., 1998). The data set used for the clustering analysis consisted of the fold changes of the RPKM values of the 154 CAZy genes (GH, CE, PL or AA9) which had significantly different fold changes in expression from the respective glucose control in at least one condition. Any zero RPKM values in this dataset were substituted with a value of 0.01 RPKM. The fold changes were log transformed and normalised with the Hierarchical Clustering module. Uncentered correlation was used as the distance measure followed by pairwise complete-linkage as the clustering method. The clustering results were visualised with the Hierarchical Clustering Viewer in GenePattern.

### 2.3 qRT-PCR for autolysis-related genes

*A. niger* was cultured in triplicate with the methods described in Section 2.1 whereby mycelia from glucose cultures were washed and transferred to either straw or media without carbon source and incubated for 6 h, 12 h, 18 h and 24 h. RNA was extracted with Trizol (Invitrogen) as described previously (Delmas et al., 2012) and the RNA was further purified and DNAse treated on-column with the NucleoSpin RNA II Kit (Machery-Nagel, Germany) according to manufacturer’s instructions. cDNA synthesis using an oligo-dT primer was performed as described previously (Delmas et al., 2012). qPCR reactions were prepared in a total volume of 10 μl using the 2X FAST SYBR-Green Master Mix with 0.25 μl of cDNA and a final primer concentration of 0.2 μM. The relative standard curve method was used where a dilution series of genomic DNA was included on each plate for quantification of the template in the cDNA samples. qPCR amplifications were carried out using the Applied Biosystems 7500 Fast Real-Time PCR system. The qPCR program used had an initial denaturation step at 95 °C for 20 s followed by 40 cycles of denaturation at 95 °C for 3 s, hybridisation and elongation at 60 °C for 30 s. The program was followed by a melt-curve step to ensure that the PCR resulted in the formation of a single product. The expression was normalised to the reference genes *actA*, An08g05910 and *sarA*, which were identified as suitable candidate reference genes for *A. niger* (Bohle et al., 2007). Supplementary Table S3 contains the primer sequences used in this study.

### 2.4 Proteome analysis

*A. niger* was grown in duplicate cultures for 6 and 9 h in AMM without carbon source and mycelium was removed from culture fitrates as described in Section 2.1. Culture filtrates were filtered through a Minisart High Flow 0.2 μm polyethersulfone filter (Sartorius Stedim Biotech, Germany) to remove particles and the proteins in 60 ml of filtrate were concentrated 3-fold using VIVA spin columns (Sartorius, Germany) with a polyethersulfone membrane and a 5000 Da MWCO. Proteins were precipitated by the addition of 6 ml of 100% (w/v) trichloroacetic acid. Proteins were collected by centrifugation (16,000*g*, 4 °C) and washed three times with cold acetone.

Samples were reduced by dithiothreitol, and alkylated by iodoacetamide, in solution before tryptic digestion (Sigma–Aldrich) at 37 °C for 16–24 h in an ammonium bicarbonate buffer. Peptides were then acidified, and subsequently desalted on C18 resin spin columns (ThermoFisher Scientific). Eluted peptides were vacuum dried before being solubilized in Switchoss Solution (0.1% (v/v) formic acid, 3% (v/v) acetonitrile). 40% of the material was injected, using a Dionex Ultimate 3000 uHPLC, onto a PepMap100 C18 2 cm × 75 μm I.D. trap column (ThermoFisher Scientific) at 5 μl min^−1^ in 0.1% formic acid, 2% acetonitrile and 35 °C in the column oven, 6 °C in the autosampler. Components in the sample were separated, over a 1 h linear gradient of increasing acetonitrile up to 72%, in 0.1% formic acid, using a 15 cm PepMap100 C18 analytical column (2 μm particle size, 10 nm pore size 75 μm I.D) (ThermoFisher Scientific) at 250 nL min^−1^ and 35 °C. The mass spectrometer analyser used was an electron transfer dissociation (ETD) enabled ThermoFisher-Scientific Orbitrap Elite, equipped with an EasySpray ESI source (ThermoFisher Scientific). Nanospray ionization was carried out at 2.0 kV, with the ion transfer capillary at 250 °C, and S-lens setting of 60%. MS^1^ spectra were acquired at a resolving power of 60,000 with an automatic gain control (AGC) target value of 1 × 10^6^ ions by the Orbitrap detector, with a range of 350–2000 m/z. Following MS^1^ analysis the top 20 most abundant precursors were selected for data dependant activation (MS^2^ analysis) using collision induced dissociation (CID), with a 100 ms activation time, and an AGC setting of 10,000 ions in the dual cell linear ion trap on normal scan rate resolution. Precursor ions of single charge were rejected, and a 30 s dynamic exclusion window setting was used after a single occurrence of an ion. The resulting spectra were searched with Mascot (Matrix Science) against the Swissprot database (with a taxonomy filter of ‘Other fungi’), and decoy database, within the Proteome Discoverer 1.3 software package (ThermoFisher Scientific). Full trypsin enzymatic specificity was required with up to 2 missed cleavages permitted. Carbamidomethylation of cysteine was specified as a fixed modification and oxidation of methionine was specified as variable modification. A mass tolerance of 5 ppm was used for precursors and 0.2 Da for fragment ions. The false discovery rates (FDRs) were set at 1% (strict) and 5% (relaxed) by Peptide Validator (workflow node within Proteome Discoverer) and were used to distribute the confidence indicators for the peptide spectral matches. Proteins required a minimum of one peptide with a 95% confidence interval or above in at least 3 biological samples in order to be reported.

### 2.5 Enzymatic activity measurements using pNP-linked carbohydrate substrates

The filtrates from glucose cultures grown for 48 h as well as from cultures with mycelium that was exposed to straw and carbon starvation for 6, 9 and 24 h as described in Section 2.1, were centrifuged to pellet solids before concentrating 15-fold using VIVA spin columns (Sartorius, Germany) with a polyethersulfone membrane and a 5000 Da MWCO. Complete Protease inhibitor cocktail (EDTA-free) (Roche) was added to the culture filtrates to protect the proteins from degradation during the concentrating process. The concentrated filtrates were divided into aliquots, flash frozen in liquid nitrogen and stored at −80 °C. Concentrated culture filtrates from duplicate flask cultures were assayed using 4-Nitrophenyl-β-d-cellobioside (pNP-Cel), 4-Nitrophenyl-α-l-arabinofuranoside (pNP-Ara), 4-Nitrophenyl-β-d-glucopyranoside (pNP-B-Glc), 4-Nitrophenyl-β-d-xylopyranoside (pNP-Xyl) and 4-Nitrophenyl-α-d-glucopyranoside (pNP-α-Glc) (all from Sigma). With assay conditions that gave a linear response between time and pNP release, up to 65 μl of the concentrated culture filtrate was assayed in a total volume of 130 μl with 2.5 mM final concentration of the substrate in 50 mM sodium acetate pH 5.0. The reactions were incubated at 37 °C for 3 h with shaking, and then stopped with 130 μl of 1 M sodium carbonate before the absorbance was measured at 405 nm with a plate reader (Biotek). The enzyme activity was expressed in pmoles pNP per minute per μl of concentrated culture filtrate assayed (pmol pNP (min μl)^−1^).

### 2.6 Enzymatic activity on polysaccharides

To detect and quantify enzymatic activities on plant-derived carbohydrates in the culture filtrates of carbon-starved cultures, filtrates of duplicate cultures were collected 2, 4, 6 and 9 h after transfer to medium without carbon source. Culture filtrates were concentrated 20-fold in the presence of protease inhibitors as described in Section 2.5. Culture filtrate (400 μl) was incubated for 6 h at 37 °C in a total volume of 1 ml with 0.05% sodium azide, 50 mM sodium acetate–citrate buffer pH 4.8 and with 0.3% (w/v) of the following substrates: sugar beet arabinan, wheat flour medium viscosity arabinoxylan, guar medium viscosity galactomannan, tamarind xyloglucan (all from Megazyme), beechwood xylan, 2-hydroxyethyl cellulose and apple pectin (Sigma Aldrich). Enzymes were inactivated through heating (10 min, 100 °C) and incubations were stored at −20 °C. Reducing sugars in the incubations were quantified using Nelson Somogyi assay method (Nelson, 1944, Somogyi, 1952) using glucose as a standard.

### 2.7 Identification of enzyme products

The filtrate of 9 h carbon-starved cultures was incubated in 2 ml final volume with arabinan, arabinoxylan, and galactomannan as detailed in Section 2.6. After termination of the reaction, the sample was filtered through a 0.2 μm filter to remove particles and 1 ml of the reaction was freeze dried. The identity of the reaction products was assessed using DASH, performing labelling of the freeze-dried material by 8-aminopyrene-1,3,6-trisulfonic acid (APTS) and separation by capillary electrophoresis generally as described elsewhere (Li et al., 2013). Reaction products where identified by comparison of migration against a library of carbohydrate standards of known identity as follows. Arabinan oligosaccharide standards were prepared from debranched arabinan (Megazyme). Mannan oligosaccharide standards DP1–DP6 were purchased from Megazyme. Arabinoxylan oligosaccharides standards were prepared from digestion of wheat flour arabinoxylan with characterised xylanases (Maslen et al., 2007). The identity of neutral monosaccharides was assessed in more detail using HPAEC-PAD essentially as described in Currie and Perry (2006) using a CarboPac PA20 column.

### 2.8 Enzymatic activity during cultivation on wheat straw

Enzymatic activities present during cultivation of *A. niger* on wheat straw, were assessed by measuring dye release from AZCL-hydroxyethyl-cellulose. Mycelium of strain AB4.1 was obtained as described in Section 2.1, and transferred to cultures containing both 1% (w/v) wheat straw and 10 mg AZCL-hydroxyethyl-cellulose (Megazyme). Culture filtrate was centrifuged for 1 min at 16000*g*, and AZCL dye release was measured by determining the OD at 560 nm. Cultivations were performed in quadruplicate and results are reported as mean ± standard deviation.

## 3 Results

### 3.1 RNA sequencing of A. niger exposed to straw or carbon starvation

We previously showed in a time course experiment of *A. niger* exposed to ball-milled wheat straw, that some genes were up-regulated between 3 and 6 h after transfer to wheat straw (named the early degradative response), while genes of the major degradative response were up-regulated later, between 6 and 9 h after transfer (Delmas et al., 2012). To expand our knowledge of the sequence of events during exposure of *A. niger* to wheat straw, we used RNA-Seq to analyse the gene transcript levels after 6 h of exposure to wheat straw, and compared these to the data obtained previously for 24 h of exposure (Delmas et al., 2012). In addition, we explored the overlap in the response to wheat straw with that to carbon starvation, by using RNA-Seq to analyse the gene expression at 6 h and 24 h of carbon starvation.

#### 3.1.1 Gene Ontology enrichment of RNA-Seq data

Gene Ontology (GO) enrichment (Ashburner et al., 2000) is a useful method to gain an overview of the changes that are occurring in a large data set. *A. niger* genes have been assigned to GO terms based on the curated GO annotation of *Aspergillus nidulans* genes (Nitsche et al., 2011). GO enrichment analysis was performed using lists of the genes with increased and decreased transcript abundance from comparisons of each of the following conditions with the respective 48 h glucose controls; 6 h straw, 6 h carbon starvation, 24 h straw or 24 h carbon starvation. Table 1 shows only the most specific hierarchical GO terms that were enriched in each of the gene lists. A full list of GO terms is available in Supplementary Table S2. GO terms related to the hydrolysis of lignocellulose polysaccharides were enriched in both the lists of genes with increased transcript abundance at 6 and 24 h straw, but more so at 24 h straw. For example, the GO term for ‘GO:0030243; cellulose metabolic process’ was enriched in the list of genes with increased transcript levels at 24 h straw but was not enriched in the list at 6 h straw. Furthermore, with regard to GO terms related to the hydrolysis of lignocellulose, ‘GO:0045490; pectin catabolic process’ was enriched in the list of genes with increased transcripts at 6 h carbon starvation and 6 h straw. At 24 h carbon starvation there was enrichment for the autolysis-related GO term ‘GO:0006032; chitin catabolic process’ indicating potential hydrolysis of the fungal cell wall. ‘GO:0009083; branched chain family amino acid catabolic process’ was enriched in the genes that had increased transcripts at 6 h carbon starvation and 24 h carbon starvation. Interestingly, ‘GO:0043938; positive regulation of sporulation’ was enriched in the genes that had increased transcripts at 24 h straw.

**Table 1:** Summary^a^ of the most specific overrepresented (FDR *q*-value <0.05) terminal node GO terms from the lists of genes with increased transcript abundance in the comparisons with the 48 h glucose control.

		6 h Carbon starvation	6 h Straw	24 h Carbon starvation	24 h Straw
*Carbohydrate metabolism*					
GO:0045490	Pectin catabolic process	+	+		+
GO:0005988	Lactose metabolic process		+		+
GO:0019566	Arabinose metabolic process		+		+
GO:0042732	d-Xylose metabolic process		+		+
GO:0045493	Xylan catabolic process		+		+
GO:0006012	Galactose metabolic process		+		
GO:0005997	Xylulose metabolic process				+
GO:0006059	Hexitol metabolic process				+
GO:0009251	Glucan catabolic process				+
GO:0010411	Xyloglucan metabolic process				+
GO:0030243	Cellulose metabolic process				+
GO:0006032	Chitin catabolic process			+	

*Amino acid metabolism*					
GO:0009083	Branched chain family amino acid catabolic process	+		+	
GO:0009073	Aromatic amino acid family biosynthetic process			+	

*Transport*					
GO:0015718	Monocarboxylic acid transport	+	+		
GO:0015850	Organic alcohol transport	+	+	+	
GO:0006559	l-Phenylalanine catabolic process			+	+

*Fatty acid metabolism*					
GO:0006631	Fatty acid metabolic process	+			
GO:0019395	Fatty acid oxidation		+		
GO:0019626	Short-chain fatty acid catabolic process		+		
GO:0046503	Glycerolipid catabolic process	+			
GO:0006633	Fatty acid biosynthetic process			+	

*Other*					
GO:0019439	Aromatic compound catabolic process	+			
GO:0019748	Secondary metabolic process	+		+	
GO:0010913	Regulation of sterigmatocystin biosynthetic process		+		
GO:0019631	Quinate catabolic process		+	+	
GO:0045733	Acetate catabolic process		+		
GO:0032787	Monocarboxylic acid metabolic process				+
GO:0043938	Positive regulation of sporulation				+
GO:0045597	Positive regulation of cell differentiation				+
GO:0072337	Modified amino acid transport				+

a See Supplementary Table S2 for the full results tables from the FetGOat GO enrichment analysis tool.

#### 3.1.2 Hierarchical clustering of genes encoding CAZymes

**Fig. 1:**
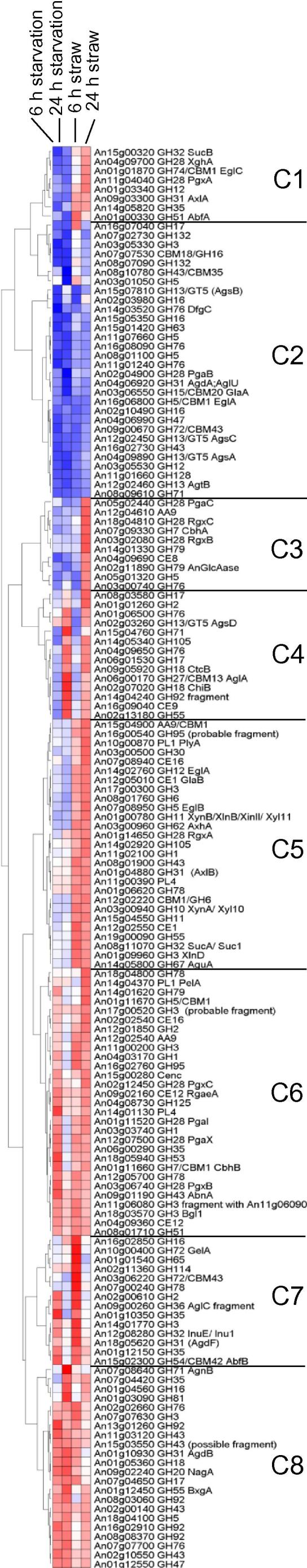
Hierarchical clustering of fold changes in CAZy genes relative to the respective glucose controls. The colouring of a cell represents the level of the normalised fold change for a gene in a particular condition relative to the glucose control; a more intense red colouration represents an increase in expression relative to the glucose control and a more intense blue colour represents a decrease in expression relative to the glucose control. C1–C8 refers to different clusters that could be resolved in the hierarchical clustering. (For interpretation of the references to colour in this figure legend, the reader is referred to the web version of this article.)

To gain a better overview of changes in expression of genes involved in lignocellulose degradation, we performed hierarchical clustering using the fold changes of the 154 (out of the total of 286) CAZy genes of the GH, CE, PL or AA9 families that had significantly different transcript abundance (adjusted *p*-value < 0.05) relative to the respective glucose control in at least one of the straw or starvation time-points (Fig. 1). Eight clusters with distinct expression patterns (C1–C8 in Fig. 1) could be resolved. Cluster C3 contains genes that only have increased transcript abundance relative to the glucose control at 24 h straw including *cbhA* and the AA9 family member An12g04610. Clusters C1 and C5 contain genes that have increased transcript abundance at both 6 h straw and 24 h straw; C5 includes xylanases such as *xynA* and *xynB.* Cluster C6 contains genes that have increased transcript abundance at 6 h starvation as well as 6 h straw and 24 h straw (and for a subset of the genes, increased transcript abundance at 24 h starvation also); C6 includes *cbhB*, as well as *pgaX* and *pgxB*, which both encode GH28 exo-polygalacturonases. For *cbhB* this expression pattern is in accordance to what has been observed previously (Delmas et al., 2012). With regard to the expression patterns in the other clusters, C4 contains genes with increased transcript abundance at 24 h starvation and 24 h straw and includes the autolytic chitinase encoding *chiB*. C7 contains genes with relatively higher transcript abundance at 6 h straw and 6 h starvation and includes the GH54 α-arabinofuranosidase encoding *abfB*. C8 contains genes with similarly increased transcript abundance across the two straw and two starvation time-points.

#### 3.1.3 Sequential gene expression during exposure to wheat straw

**Fig. 2:**
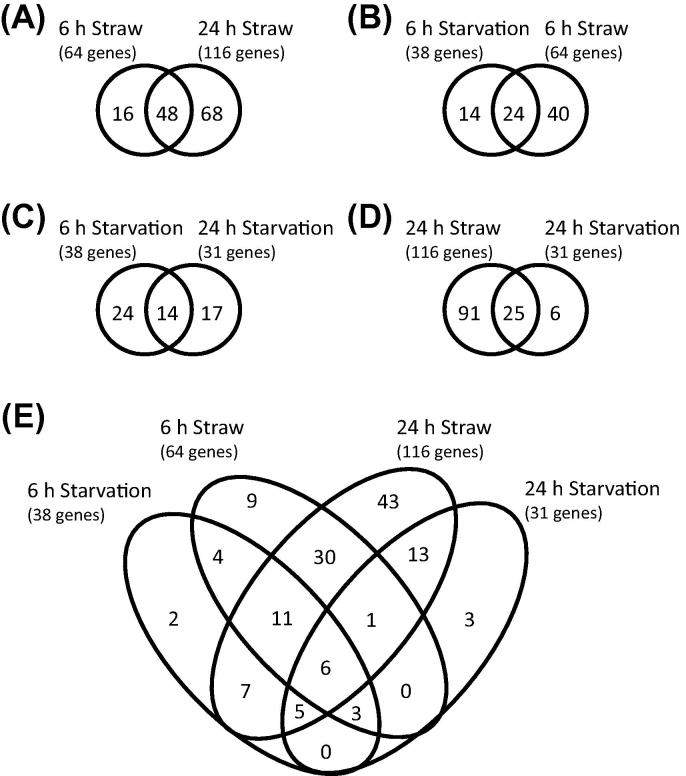
Venn diagrams showing the number of genes with increased transcript levels in the conditions 6 h and 24 h straw (A), 6 h straw and 6 h carbon starvation (B), 6 h and 24 h starvation (C), 24 h straw and 24 h starvation (D) and all 4 conditions (E).

Of the 436 carbohydrate active enzymes and associated modules as found in the CAZy database to be currently annotated in the *A. niger* genome (Cantarel et al., 2009), 132 CAZyme-encoding genes had raised transcript levels during growth on straw on at least one time-point, when compared to growth on glucose. Increased transcript levels were detected for 64 genes after 6 h of exposure to straw and for 116 genes after 24 h of exposure (Fig. 2A). Transcript levels of 48 of the CAZyme-encoding genes were increased at both time-points. These numbers indicate that there is a substantial difference in the response to straw after 6 and 24 h, with a much larger number of CAZyme-encoding genes expressed at the 24 h time-point. This observation fits with the sequential induction of a number of genes encoding CAZymes that we observed previously (Delmas et al., 2012).

#### 3.1.4 The early response to wheat straw

For 16 CAZyme encoding genes an increase in transcription was detected after 6 h of exposure to wheat straw, but not after 24 h (Fig. 2A, Table S4). Of these, 4 genes encoded enzymes active on β-1,3-glucan in the fungal cell wall. There were 7 genes that also showed increased levels of transcripts after 6 h of carbon starvation and these are discussed in Section 3.1.6.

A set of 48 genes had increased transcript levels after both 6 h and 24 h exposure to wheat straw, compared to glucose conditions (Table S4). The gene set included a high proportion of genes that encode enzymes with (predicted) activity on hemicelluloses. There were 9 genes predicted to encode enzymes with activity on the arabinoxylan backbone, including *xynA*, *xynB* and An15g04550, which encode endo-1,4-beta-xylanases of GH10 and GH11 families, and 2 putative β-xylosidase encoding genes. Also included in this category were genes encoding enzymes active on backbone decorations; *axhA* encoding a GH62 arabinoxylan-specific arabinofuranosidase, *aguA* encoding a α-glucuronidase, and the acetyl xylan esterase and feruloyl esterase encoding *axeA* and An12g02550 respectively. Furthermore, 5 genes that encode enzymes with putative activity on xyloglucan also had increased transcript levels. These were An01g03340, which encodes a GH12 xyloglucan specific endo-β-1,4-glucanase, as well as 2 genes that encode GH31 α-xylosidases AxlA and AxlB, and 2 genes that encode GH95 α-fucosidases.

In addition to the broad range of genes encoding hemicellulases, a subgroup of genes encoding cellulolytic and pectinolytic genes showed increased transcript levels after both 6 and 24 h of exposure to wheat straw. These include An12g02220, which encodes a GH6 cellobiohydrolase, as well as the gene encoding GH5-5 endo-β-1,4-glucanase EglB that is active on both xyloglucan and cellulose.

A second major feature in the set of genes that had increased transcript levels both after 6 h and 24 h of exposure to wheat straw, is that 17 genes encoded enzymes active on plant polysaccharides, and also have increased transcript levels after 6 h of carbon starvation (Table 2). The expression profile of this set of genes suggests that these genes make up the early degradative response of *A. niger* to wheat straw. This important set of genes is discussed in more detail below in Section 3.1.6.

**Table 2:** Genes with increased transcript levels after 6 h exposure to wheat straw and 6 h carbon starvation.

		CBS 513.88	ATCC1015	Description	CAZy module(s)	Predicted substrate^a^	Gene	RPKM^b^						Fold change^c^			
								Glu	6 h Straw	6 h No C	24 h No C	Glu	24 h Straw	6 h Straw	6 h No C	24 h No C	24 h Straw
*6 h Starvation + 6 + 24 h straw*																	
	Cellulose	An11g00200	179265	Putative β-glucosidase precursor	GH3	Cellulose		0.7	304.4	8.2	1.9	0.5	332.1	459.7	12.4	2.9	633.6
		An18g03570	56782	β-Glucosidase	GH3	Cellulose	bgl1/bglA	0.8	178.9	33.4	13.1	1.1	97.5	215.9	40.3	15.8	80.4
		An04g03170	131747	Putative β-glucosidase precursor	GH1	Cellulose		0.0	3.7	0.3	0.0	0.0	1.5	N/A	N/A	N/A	202.2
		An03g03740	213437	β-Glucosidase	GH1	Cellulose		1.2	4.7	5.4	1.1	2.5	28.8	3.9	4.5	0.9	10.5
	Pectin	An01g12150	51764	β-Galactosidase	GH35	Pectin	lacA	0.2	46.3	11.1	0.5	0.3	17.0	189.2	45.4	2.0	60.0
		An09g01190	203143	Endo-α-1,5-l-arabinase	GH43	Pectin	abnA	0.0	9.9	37.9	1.4	0.0	26.8	841.8	3209.2	118.7	1141.6
		An15g02300	200605	α-l-Arabinofuranosidase B	GH54-CBM42	Pectin, xylan	abfB	0.1	9.9	4.1	0.1	0.1	2.0	189.9	78.3	2.0	19.0
		An08g01710	38549	α-l-Arabinofuranosidase	GH51	Pectin	abfC	0.2	9.6	2.5	1.3	0.2	12.8	55.9	14.9	7.3	69.2
		An15g03550	182100	Putative α-l-arabinofuranosidase	GH43	Pectin		0.0	5.2	4.4	8.4	0.1	16.8	236.9	200.1	382.3	163.4
		An02g10550	197735	Putative endo-α-1,5-arabinanase	GH43	Pectin	abnC	0.1	3.8	18.3	22.1	0.1	22.3	26.2	125.5	151.2	149.6
		An06g00290	177434	β-Galactosidase	GH35	Pectin	lacC	0.1	2.2	2.2	0.1	0.2	14.8	22.8	22.9	1.1	55.7
		An12g07500	42184	Exo-polygalacturonase X	GH28	Pectin	pgaX	0.1	2.1	1.7	0.2	0.4	7.9	14.4	11.5	1.0	15.4
		An03g06740	191158	Exo-polygalacturonase B	GH28	Pectin	pgxB	0.0	1.6	2.3	0.1	0.0	10.0	N/A	N/A	N/A	236.5
		An04g09360	51400	Putative rhamnogalacturonan acetyl esterase	CE12	Pectin	rgaeB	0.1	1.2	0.7	0.5	0.4	5.8	11.1	6.7	4.6	13.2
	Other	An12g08280	56664	Exo-inulinase	GH32	Inulin	inuE	0.0	11.4	0.4	0.0	0.0	0.2	1785.8	62.8	3.1	13.4
		An01g12550	205517	Mannosyl-oligosaccharide 1,2-α-mannosidase	GH47	Glycans	msdS	0.6	3.7	8.0	14.4	0.5	16.7	6.2	13.6	24.3	29.8
		An18g04100	202490	Putative exo-β-1,3-glucanase;	GH5	β-1,3-Glucan	exgA	0.3	2.3	4.5	21.0	0.1	31.9	8.8	17.3	81.3	197.9

*6 h Starvation + 6 h straw*																	
		An01g05360	170148	Putative chitinase, group A	GH18	Chitin	cfcD	0.3	2.8	2.8	6.1	1.3	3.9	10.4	10.1	22.3	3.0
		An18g02690	131352	Dihydrogeodin oxidase	AA1		mcoI	0.1	1.7	0.5	1.1	0.3	0.9	22.3	6.9	13.6	2.9
		An07g04650	53278	Putative β-1,3-glucanotransferase, generating β-1,6-linkages	GH17	β-1,3-Glucan	bgtC	0.9	4.0	2.7	3.9	1.2	2.4	4.7	3.1	4.5	2.1
		An01g10350	46429	β-Galactosidase	GH35	Pectin	lacB	0.3	2.2	2.7	0.1	0.3	0.7	8.6	10.2	0.5	2.0
		An09g00260	212736	α-Galactosidase	GH36	Galacto(gluco)mannan	aglC	0.5	31.9	4.7	0.5	1.3	2.9	63.1	9.3	0.9	1.9
		An01g10930	119858	Putative α-glucosidase	GH31	Starch	agdB	8.0	44.6	36.4	74.6	10.4	15.2	5.6	4.6	9.3	1.4
		An01g02730	N/A	Putative chitin-peptidoglycan binding protein	CBM50-CBM50	Chitin, peptidoglycan		0.0	0.9	0.6	0.2	0.0	0.0	N/A	N/A	N/A	1.9

a Please note that encoded exo-acting proteins can have multiple substrates.

b Expression values are rounded to one decimal.

c Fold changes that are significantly (FC ⩾ 3.0 & *p* < 0.05) different from glucose conditions are underlined. Genes with fold changes listed as N/A have an expression level of 0 RPKM in glucose conditions.

#### 3.1.5 The late response to wheat straw

A noticeable feature in the 68 CAZyme-encoding genes with increased transcript levels observed only after 24 h exposure to wheat straw, and not after 6 h of exposure (Table S5), is that genes with the highest expression values encode enzymes that are active on cellulose. These include GH7 and GH6 cellobiohydrolase-encoding *cbhA* and An08g01760, and the GH12 and GH5–5 cellulase-encoding *eglA* and An01g11670. Also, AA9 lytic polysaccharide monooxygenase-encoding genes showed increased expression after 24 h exposure to wheat straw. It would therefore appear that the full cellulolytic arsenal is expressed as part of the late-onset major degradative response. To confirm that the cellulolytic response was initiated relatively late, the cellulolytic activity in wheat straw cultures was assessed by detection of AZCL-hydroxyethyl-cellulose degradation. After 6 h of cultivation, no activity was detected against the substrate (OD_590_ of 0.00 ± 0.01), after 12 h the activity was low (0.03 ± 0.01), and after 24 h the activity had increased substantially to 0.37 ± 0.29. The late onset of AZCL-hydroxyethyl-cellulose degradation activity thus fits with the expression profiles of cellulolytic genes.

Interestingly, although wheat straw contains only a low amount (∼10%) of pectin (Caffall and Mohnen, 2009, Carpita, 1996), a broad range of genes encoding enzymes with pectinolytic activities was expressed only after 24 h exposure to wheat straw. The expression level of these genes remained relatively low (<32 RPKM) when compared to the induced cellulolytic genes (0.4–1508 RPKM). Genes with increased transcript levels encode rhamnogalacturonan I backbone-degrading enzymes such as putative exo-rhamnogalacturonases RgxA and RgxB the rhamnogalacturonan acetyl esterase RgaeA (Martens-Uzunova and Schaap, 2009) and two GH78 putative α-l-rhamnosidases that may have activity against terminal non-reducing rhamnose in rhamnogalacturonan (Mutter et al., 1994). Notably, no genes related to degradation of rhamnogalacturonan I side chains had substantially raised transcript levels only after 24 h exposure to wheat straw. The genes encoding homogalacturonan-degrading pectate lyase PlyA, pectin lyase PelA endo-polygalacturonases PgaI and PgaC, putative acetylesterase PaeA (An02g02540) and putative pectin methylesterase PmeB (Martens-Uzunova and Schaap, 2009) were also induced only after 24 h exposure to wheat straw, as were all 3 genes in the *A. niger* genome predicted to encode enzymes active on xylogalacturonan (Martens-Uzunova and Schaap, 2009), i.e. endo-xylogalacturonan hydrolase-encoding *xghA* and exo-polyxylogalacturonases encoding *pgxA* and *pgxC*. The timing of this late, broad pectinolytic response contrasts with the early hemicellulolytic response; 4 genes encoding hemicellulases showed an increase in transcript levels only after 24 h of exposure to wheat straw whereas transcription of the majority of the hemicellulose genes was already increased after 6 h of exposure to wheat straw.

In summary, when comparing gene expression after 6 h and 24 h of exposure to wheat straw, the most noticeable changes are: (1) genes encoding hemicellulases showed increased transcript levels as early as 6 h. The increase in transcript levels of most of these genes continued in time, being much higher at 24 h than at 6 h. (2) Transcript levels of most genes encoding enzymes acting on cellulose and the pectin backbone, were increased later, after 24 h of exposure to wheat straw. (3) Genes with increased transcript levels during carbon starvation comprised a considerable part of the genes identified during exposure to wheat straw.

#### 3.1.6 Carbon starvation response overlaps with response to wheat straw

The RNA-Seq data showed that 38 CAZyme-encoding genes had significantly higher transcript levels after 6 h of carbon starvation compared to growth on glucose. Of these 38 genes, 24 also exhibited increased transcript levels after 6 h exposure to wheat straw (Fig. 2B, Table 2). And, of these, 17 genes also showed increased transcript levels after 24 h of exposure to wheat straw. Importantly, of the 24 genes that had increased transcript levels after 6 h under both in the carbon starvation conditions and straw conditions, most (18) were predicted to be active on plant-derived carbohydrates.

A high proportion of these genes encoded putative pectin-acting enzymes, especially those acting on arabinan or arabinogalactan side chains of rhamnogalacturonan I. These included the genes encoding endo-l,5-α-l-arabinase AbnA, putative endo-l,5-α-l-arabinas AbnC and a third GH43 enzyme, which were induced >125-fold during carbon starvation. Also, the genes encoding β-galactosidases LacA, LacB and LacC, and α-arabinofuranosidases AbfB (GH54) and AbfC (GH51) showed raised transcript levels during both early carbon starvation and exposure to straw. These enzymes could be active on arabinan but also on arabinoxylan or xyloglucan (van den Brink and de Vries, 2011). Also, genes encoding exo-acting enzymes active on the rhamnogalacturonan I and homogalacturonan backbones were identified in this gene set, such as *pgaX* and *pgxB*, which encode GH28 exo-polygalacturonases.

In conclusion, the CAZy enzymes encoding genes that show increased transcript levels during both early (6 h) carbon starvation as well as after short (6 h) exposure to wheat straw, mainly encode enzymes that are active on plant-derived carbohydrates, and include a large proportion of enzymes predicted to be active on relatively easily accessible substrates such as rhamnogalacturonan I side chains, hemicellulose or cellulose oligosaccharides. These genes are part of the early degradative response, and encode enzymes that could have a ‘scouting’ role during lignocellulose degradation.

#### 3.1.7 Early versus late carbon starvation

After 6 h of carbon starvation, 38 CAZy genes had increased transcript abundance while after 24 h there were 31 CAZy genes with increased transcript levels. There was an overlap of 14 CAZy genes (Fig. 2C, Table S6). As with the response to wheat straw, there were considerable differences between the early (6 h) and late (24 h) response to carbon starvation. The early response to carbon starvation included a large proportion (at least 18 out of 31) of genes encoding CAZy enzymes thought to be active in the scouting response (described above). However, only 4 of these genes also showed increased transcript levels after 24 h of carbon starvation. Of the CAZy genes that had increased transcript levels after 24 h of carbon starvation, 16 genes were possibly involved in autolytic fungal cell wall degradation or remodelling (Nitsche et al., 2012) while only 4 of these (An07g07700, *exgA*, *cfcD* and *bgtC*) were identified both after 6 and 24 h, suggesting that there is a greater autolytic response at 24 h starvation compared to 6 h starvation. This is consistent with the enrichment of the relevant GO term ‘GO:0006032: chitin catabolic process’ (FDR *q* value <0.05) at 24 h starvation, but not at 6 h starvation. However, even at 24 h starvation, the progression towards recycling of cellular components seems incomplete; there was a lack of enrichment for autophagy-related GO terms (see Supplementary Table S2).

Interestingly, 13 genes that were expressed after 24 h of carbon starvation, but not after 6 h, are also expressed to high levels after 24 h exposure to wheat straw (Table S6, Fig. 2D and E). These include the genes encoding the fungal autolysis-related GH18 chitinase CfcA/ChiB (Yamazaki et al., 2007) and GH81 endo-β-1,3-glucanase (Szilagyi et al., 2010), as well as other putative fungal cell wall active β-1,3-glucanases. The same expression pattern applied to genes that are strongly associated with sporulation, such as *ctcB* encoding a GH18 conidiophore specific chitinase (van Munster et al., 2013) and *brnA* encoding an AA1 putative multicopper oxidase, which is involved in production of spore pigmentation (Jorgensen et al., 2011). In summary, we found that genes encoding enzymes with a role in the scouting response during lignocellulose degradation, showed increased transcript levels mainly during early carbon starvation. The early response lacked significant increases in transcript abundance of many autolysis-related genes and lacked enrichment of relevant GO terms compared to the response at 24 h starvation. This is of interest as previous studies of autolytic fungal cell wall degradation have described this process only from the point where autolytic hydrolases were up-regulated (Nitsche et al., 2012, Szilagyi et al., 2013). For example, one study using *A. nidulans* showed that transcripts for autolysis-related genes *chiB* and *nagA* increased significantly after 4 h in carbon starvation conditions (Szilagyi et al., 2013). We therefore investigated the up-regulation of genes encoding fungal cell wall acting enzymes in more detail.

### 3.2 qRT-PCR of transcripts relevant to autolysis

**Fig. 3:**
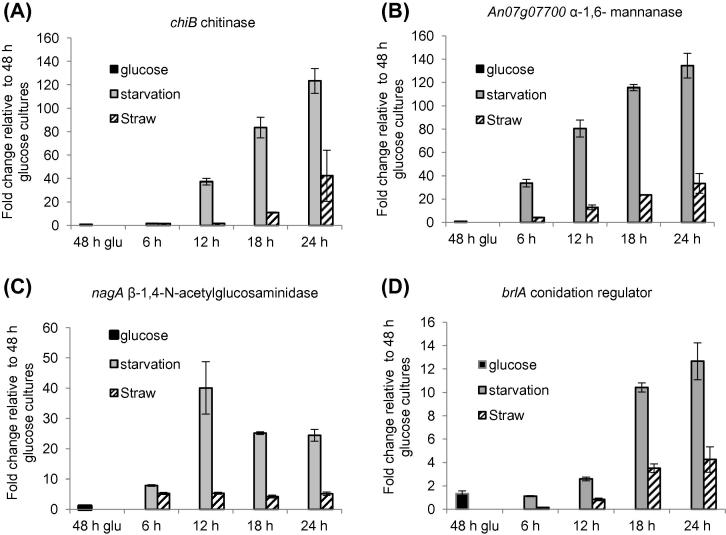
qRT-PCR expression analysis of autolysis related genes in *A. niger* on straw and carbon starvation. The expression for each gene is relative to the expression of that gene in the glucose control condition. The expression of the genes was normalised to the expression of three reference genes (*sarA*, *actA* and *An08g05910*). *chiB*: chitinase (A), *An07g07700*; α-1,6-mannanase (B), *nagA*: β-1,4-N-acetylglucosaminidase (C) and *brlA*: conidation regulator (D).

To investigate if the transcript abundance of the autolysis related genes increased during starvation and if the transcript levels of the genes were increased to the same extent on straw as on starvation over time, we followed transcription of 4 genes in time by qRT-PCR with more time-points (12 h and 18 h) than were available for the RNA-Seq study (6 h and 24 h). A subset of genes (*chiB*, *nagA*, *An07g07700* and *brlA*) was chosen based on a mixture of patterns in their transcript abundance in the RNA-Seq data (Fig. 3). When measured by qRT-PCR, the increase in transcript abundance (>3-fold increase in expression compared to glucose control and *P* < 0.05 (Student’s *t*-test)) in starvation conditions occurred for *chiB* and *brlA* after 18 h starvation whereas transcripts for *nagA* and *An07g07700* were already increased at 6 h starvation. The size of the increase in transcripts was generally larger in carbon starvation conditions than in straw conditions (Fig. 3). For example, at the 12 h time-point, *chiB*, *nagA* and *An07g07700* had 21-fold, 7-fold and 6-fold higher transcript levels respectively during carbon starvation compared to the straw conditions.

### 3.3 Carbon-starved cultures secrete CAZymes

The transcriptome analysis of early carbon starving cultures indicated the up-regulation of genes encoding CAZymes that are active on plant carbohydrates. To determine whether this increased gene transcription results in the secretion of enzymes, the proteome of carbon starving cultures was analysed at 6 h and 9 h of carbon starvation. A total of 8 CAZymes were identified reliably in the culture filtrates, 6 of which were plant-polysaccharide degrading enzymes. Of these, endo-1,5-arabinase AbnC, and cellobiohydrolase CbhB were encoded by genes that also showed increased transcript levels during carbon starvation and after early (6 h) exposure to wheat straw. Glucoamylase GlaA and α-glucosidase AgdA were encoded by genes that were expressed with high transcript levels during growth on glucose as well as under carbon starvation. The α-galactosidases AglA and AglB were also identified, although they had low gene transcript levels during carbon starvation. Except for endo-1,5-arabinase AbnC, the identified enzymes have exo-acting activity. A number of proteins were identified only once, these include arabinofuranosidase AbfB, GH11 xylanase XynB, β-galactosidase LacA and β-1,4-mannanase Man5A. The reason behind the identification of such a small number of proteins may be the inherent difficulty of detecting proteins at a low concentration, or may be related to the carbon starvation induced up-regulation of proteolytic activities. The identified set of proteins together makes up a flexible mix of activities, which may be active on rhamnogalacturonan I side chains, (arabino)xylan, β-1,4-glucan and galactomannan.

### 3.4 Enzymes in filtrates of carbon-starved cultures have activity towards pNP substrates

**Fig. 4:**
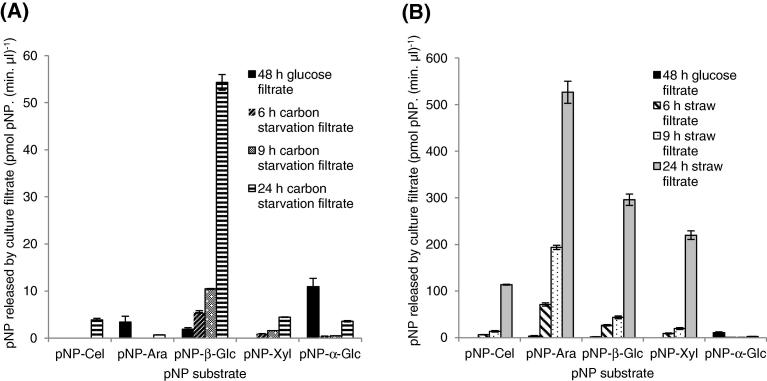
Enzyme activity towards pNP substrates from culture filtrates from various conditions. (A) Activity towards pNP substrates in filtrates from carbon starvation and glucose control cultures, and (B) from straw and glucose control cultures. The data from straw cultures and carbon starvation cultures are shown on separate graphs because the level of activity is different. In both (A) and (B), the same glucose control data are shown. Abbreviations are: pNP-Cel: 4-Nitrophenyl-β-d-cellobioside; pNP-Ara: 4-Nitrophenyl-α-l-arabinofuranoside; pNP-β-Glc; 4-Nitrophenyl-β-d-glucopyranoside; pNP-Xyl: 4-Nitrophenyl-β-d-xylopyranoside; pNP-α-Glc; 4-Nitrophenyl-α-d-glucopyranoside.

The RNA-seq showed that there were transcripts for CAZy enzymes at higher levels in *A. niger* in the carbon starvation conditions and proteome data confirmed the presence of CAZymes. To test whether this led to enzyme activity in the culture filtrates, the activities were assayed with pNP-labeled substrates. Filtrates from *A. niger* cultured with wheat straw were also assayed to compare to the levels of activity in the culture filtrates from the carbon starved cultures. Activity was detected in culture filtrates from 6, 9 and 24 h carbon starving cultures towards pNP-β-d-glucopyranoside and pNP-β-d-xylopyranoside demonstrating the presence of β-glucosidase and xylosidase activity respectively in the filtrates from carbon starving cultures (Fig. 4A). No activity could be detected towards pNP-β-d-cellobioside or pNP-α-l-arabinofuranoside. In contrast, enzyme activity from the straw culture filtrates was detected towards each of the four substrates (Fig. 4B). For each of these four substrates, the activity increased with the length of time *A. niger* was cultured with the straw (Fig. 4B). As expected, the activity towards pNP-α-d-glucopyranoside was lower in culture filtrates from the straw and carbon starving cultures compared to the filtrate from the glucose cultures (Fig. 4).

### 3.5 Enzymes in filtrates of carbon-starved cultures have activity towards complex plant polysaccharides

The enzymatic activity in the filtrate of cultures starving for up to 9 h was investigated further to determine activity against complex plant-derived carbohydrates. Filtrates of cultures starving for 2, 4, 6 and 9 h were incubated with a range of plant polysaccharides. Reducing sugar end measurements showed that at all of these time-points the culture filtrates had activity against apple pectin, beechwood xylan and wheat arabinoxylan (Fig. S1). Furthermore, very low levels of activity against guar galactomannan, sugar beet arabinan and 2-hydroxyethyl cellulose were also detected. No activity was detected against tamarind xyloglucan. This indicates that the enzymes in the filtrate of carbon-starving cultures are not only active against simple pNP-labelled substrates, but also against complex plant-derived polymers. The levels of activity between the time-points were similar, suggesting that the secretion of active enzymes by carbon starving cultures may be a rapid response.

The proteome analysis and enzyme activity measurements show that CAZymes with plant polysaccharide degrading activity are secreted during carbon starvation. To test whether these CAZymes may release known or potential inducers for gene transcription, we investigated the identity of the products that were generated by the activity of these enzymes on a number of plant polysaccharides.

**Fig. 5:**
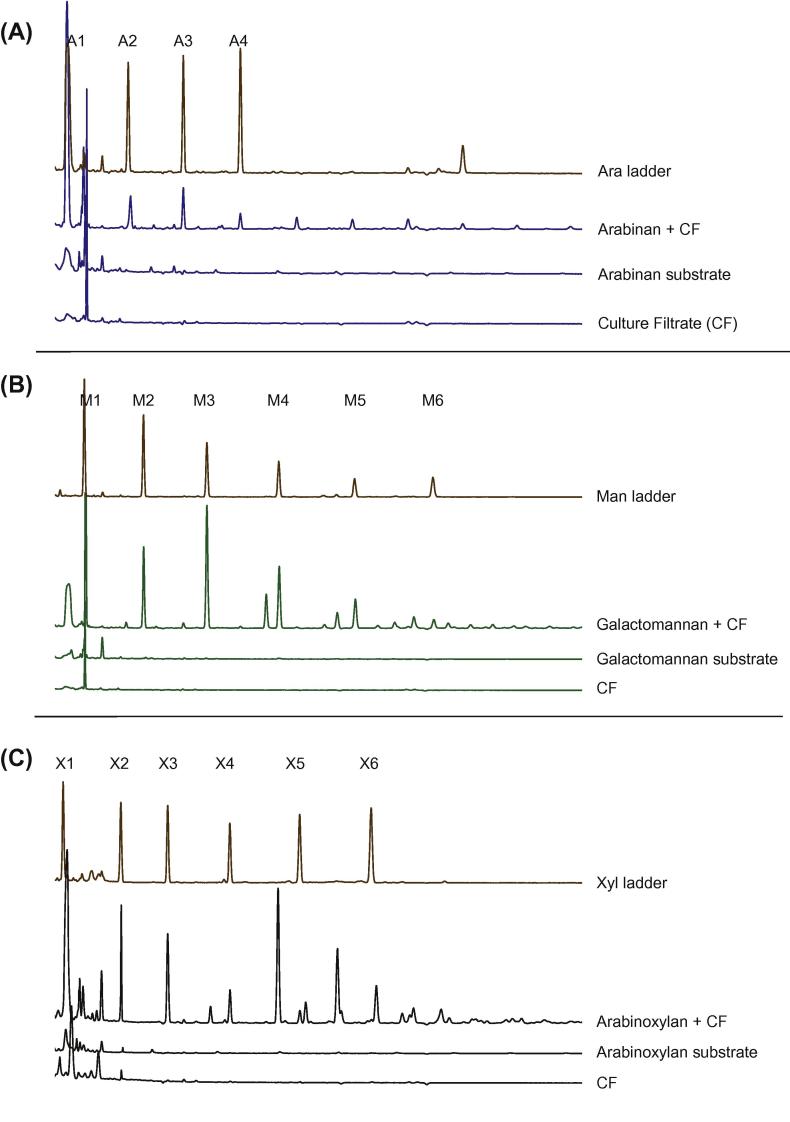
Separation of enzyme products generated by carbon starved culture filtrates incubated with arabinan (A), galactomannan (B) or arabinoxylan (C) using DASH. CF; culture filtrate, Ara; arabinan oligosaccharides, Man; mannan oligosaccharides, Xyl; xylose oligosaccharides. Numbers 1–6 indicate length of the oligosaccharide in the ladder.

DASH analysis of reaction products of the 9 h starvation culture filtrate incubated with arabinan showed oligosaccharides are released with a degree of polymerisation (DP) in the range of DP1–DP10 (Fig. 5A). Products released from galactomannan included mannan oligosaccharides of at least up to DP5, as well as additional peaks next to DP4 and DP5 which are likely to be galactose decorated mannan oligosaccharides (Fig. 5B). A range of oligosaccharides were released from arabinoxylan. Peaks with similar mobility to linear xylan oligosaccharides DP2–DP5 were found as well arabinose-decorated xylose oligosaccharides of DP3 and larger (Fig. 5C).

Further analysis of the carbohydrate monomers released from wheat arabinoxylan with HPAEC–PAD showed that arabinose was produced, indicating the presence of xylan arabinosidase activity in the culture filtrates (Fig. S2). No xylose was detected after incubating the culture filtrate with arabinoxylan. l-Arabinose is an inducer for the arabinolytic system in *A. niger* (Seiboth and Metz, 2011), via the transcriptional regulator AraR (Battaglia et al., 2011). Detection of this molecule among the reaction products thus confirms that the CAZy enzymes that are produced during carbon starvation can convert plant polymers to produce carbohydrates that are known to induce the expression of a set of CAZymes in *A. niger*.

## 4 Discussion

When saprobic fungi such as *A. niger* are exposed to complex lignocellullosic substrates, their response is complex and leads to the up-regulated transcription of a broad array of CAZyme-encoding genes and the secretion of both enzymes and accessory proteins. Despite recent progress, knowledge on the order of events, timing and regulatory mechanism behind this response remains limited (Glass et al., 2013). We show in this study that the response of *A. niger* to wheat straw is sequential, and that carbon starvation plays a major role in the induction or initial response of the fungus.

Our results show that in response to wheat straw, the transcriptome of *A. niger* changes gradually. The majority of genes that have increased transcription levels after 6 h make up a sub-population of those with increased transcription levels after 24 h. After 6 h the response includes genes encoding hemicellulolytic enzymes such as those predicted to be active on arabinoxylan and xyloglucan. In addition, 5 genes encoding cellulolytic enzymes were up-regulated. These genes encoded a GH6 cellobiohydrolase and GH5-5 endo-β-1,4-glucanase EglB, which have a family 1 carbohydrate-binding module, CBM1. Also, the early response gene *cbhB*, identified previously (Delmas et al., 2012), encodes a GH7 cellobiohydrolase with a CBM1. The CBM1 can assist enzyme function by promoting association to the substrate (Boraston et al., 2004, Herve et al., 2010), thus it is possible that cellulolytic genes with a CBM1 could function in the initial attack of the cellulose, making the substrate more accessible or releasing inducers for other cellulolytic enzymes.

The early up-regulation of the xylanolytic genes could be related to the early release of xylose, arabinose, and (arabino)xylan oligosaccharides from the substrate. Alternatively, it could be related to the presence of low amounts of xylose in the cultivation medium containing ball-milled wheat straw. However, carbohydrate analysis of culture filtrates of typical straw cultures showed that there was only ∼30 μM xylose and ∼10 μM arabinose present before inoculation (Delmas et al., 2012). After 12 h of cultivation, this increased to ∼70 and ∼40 μM respectively (Delmas et al., 2012), consistent with the early induction of enzymes capable of releasing these monomers. Regulators XlnR and AraR are responsible for up-regulation of such genes in *A. niger*. Where the xylose responsive regulator XlnR is conserved in most filamentous ascomycetes, AraR is a Eurotiales-specific l-arabinose responsive activator of genes encoding l-arabinose releasing enzymes (Battaglia et al., 2011). The minimum concentration of arabinose or xylose that is sufficient to result in induction of XlnR- and AraR-regulated genes is unknown. However, the lowest concentration of xylose tested in *A. niger* was 1 mM, which was sufficient to cause the maximum induction of most tested xylanolytic genes (*aguA*, *faeA*, *xynB*, *xynA*, *xlnD*, *lacA estA*, *axeA*) 1 or 2 h after induction, as well as that of a selection of cellulolytic genes (*bglA*, *eglB*, *eglA)* (de Vries et al., 1999, Mach-Aigner et al., 2012). Except for *eglA*, these genes also had increased transcription levels after 6 h exposure to wheat straw. We detected that the culture filtrates of carbon starved cultures contain enzymatic activities that release (arabino)xylan oligosaccharides from plant material such as wheat straw. The up-regulation of xylanolytic enzymes after exposure to wheat straw could therefore be caused by the release of such oligosaccharides.

Over a third of the genes that had increased transcript levels at both 6 and 24 h after exposure to wheat straw, also had increased levels after 6 h of carbon starvation. The transcriptional response to carbon starvation thus appears to contribute a substantial part of the early response to lignocellulose. We showed previously that de-repression of carbon catabolite repression is responsible for the low-level induction of three genes encoding CAZy enzymes (Delmas et al., 2012). Other authors have also shown that carbon starvation or deletion of *creA* results in the up-regulation of several plant-hydrolytic enzymes in *A. niger* (Martens-Uzunova and Schaap, 2009, Ruijter et al., 1997). In addition, regulators involved in the carbon starvation response, such as *A. nidulans* XprG, AtmA, SnfA or SchA (Brown et al., 2013, Katz et al., 2013, Krohn et al., 2014) may play a role in the response to carbon starvation and the early response to lignocellulose degradation. We have now identified the genes that have increased transcript levels under both carbon starvation and early lignocellulose degradation. They mainly encode enzymes that are active on arabinan or arabinogalactan side chains of pectin, as well as (arabino)xylan and galacto(gluco)mannan. The enzymes encoded by these genes have been suggested to be responsible for release of small inducers from substrates in the environment of the fungus, acting either alone or together with enzymes encoded by constitutively expressed genes (Glass et al., 2013, Martens-Uzunova and Schaap, 2009). We show here, using enzymatic activity measurements as well as proteomics, that culture filtrates of carbon-starved cultures indeed contain enzymes that are active on plant polysaccharides. These enzymes include those encoded by constitutively expressed genes as well as those up-regulated by carbon starvation, which is consistent with a model where both types of enzymes contribute to the release of inducing molecules. This mix of secreted enzymes is capable of degrading several complex polysaccharides such as arabinan, galactomannan and (arabino)xylan (Fig. 5), and even wheat straw (results not shown), resulting in the release of a range of oligosaccharides and monomers. A few of these products have been shown to induce expression of CAZy enzymes acting on plant-polysaccharides. In *T. reesei* cultures growing on glycerol, addition of mannobiose induced *abf1*, which encodes an alpha-arabinofuranosidase, as well as *agl1* and *agl2*, which encode alpha-galactosidases. Addition of xylobiose induced expression of xylanolytic enzymes (*xyn1* and *xyn2*, which encode β-xylanases, and gene *bxl1*, which encodes a β-xylosidase). Growth on arabinose/arabitol or galactose was sufficient to induce *abf1* and *bxl1* or *agl1* and *agl2* respectively (Margolles-clark et al., 1997). l-Arabinose and its downstream pathway intermediate l-arabitol were also identified as inducers for the arabinolytic system in *A. niger* (Seiboth and Metz, 2011). These data indicate that increased gene transcription levels, found during carbon starvation, could indeed eventually lead to the generation of inducers from complex substrates found in the direct environment of the carbon starved fungus.

During the later (24 h), major degradative response to wheat straw, a greater number of CAZy enzyme-encoding genes showed increased transcription levels when compared to 6 h exposure to wheat straw, these encoded mainly cellulolytic and pectinolytic enzymes. Comparison of the late, major degradative response to straw to prolonged (24 h) carbon starvation, showed that genes encoding enzymes that are involved in autolytic degradation of the fungal cell wall, exhibit increased transcript levels under both conditions. Also, genes associated with conidiation (*brlA*, *ctcB*) were expressed in both of these conditions, suggesting that the fungus experiences carbon starvation during later wheat straw degradation. Both RNA-Seq data and qRT-PCR data confirm that transcript levels of such genes increased during later carbon starvation. Thus, the fungal response to carbon starvation changes over time.

We conclude from this study that the model proposed previously to describe the responses of *A. niger* to wheat straw (Delmas et al., 2012) can be refined. Our data show that early-induced genes upon exposure to wheat straw overlap with those that are induced by carbon-starvation conditions, and support the suggestion that alleviation of carbon catabolite repression may be (partly) responsible for their induction. There is a succession of transcriptional events over time when *A. niger* is exposed to wheat straw, or is starved of carbon, that leads to the secretion of proteins with roles in the saccharification of lignocellulose. Sugar-based compounds were detected that may serve as inducers of genes expressed at later times.

## Appendix A Supplementary material

Supplementary data 1 Supplementary Table S1. RNA-Seq expression values for all genes.

Supplementary data 2 Supplementary Table S2. Lists of the genes used for the GO enrichment analysis along with a full list of GO terms from the FetGOat analysis tool from the analysis of the overrepresented GO terms in the Biological Process (BP) or Molecular Function (MF) ontologies. These lists of GO terms were generated with the FetGOat tool by setting the critical FDR *q*-value to 1 so as to show also what the FDR *q*-value was of GO terms that were not enriched at the at the critical FDR *q*-value threshold of 0.05.

Supplementary data 3 Supplementary Table S3. Sequences of primers used in this study.

Supplementary data 4 Supplementary Table S4. Genes with increased transcript levels after exposure to wheat straw for 6 h or both 6 h and 24 h.

Supplementary data 5 Supplementary Table S5. Genes with increased transcript levels after 24 h but not 6 h exposure to wheat straw.

Supplementary data 6 Supplementary Table S6. Genes with increased transcript levels after 6 and/or 24 h of carbon starvation.

Supplementary data 7 Supplementary Table S7. CAZymes identified by proteome analysis.

Supplementary data 8 Supplementary Fig. S1. Enzyme activity in filtrates of carbon starved cultures. Activities against plant-derived carbohydrates, as determined by reducing carbohydrate end measurements, of incubations with filtrate of cultures starving for 2 h (white), 4 h (grey), 6 h (hashed), and 9 h (black), given as mean ± standard deviation.

Supplementary data 9 Supplementary Fig. S2. HPAEC-PAD analysis of reaction products of enzyme activity against arabinoxylan in filtrates of carbon-starved cultures. The carbohydrate monomers present in the culture filtrate (A) were compared to those generated by incubation of culture filtrate with arabinoxylan (B). Identification of the reaction product as arabinose was confirmed by spiking the reaction mixture with arabinose (C). Peaks were identified by comparison to a monosaccharide standard (D) containing fucose (Fuc), rhamnose (Rha), arabinose (Ara), galactose (Gal), glucose (Glc), xylose (Xyl) and mannose (Man).
