# Supplementary material for: The role of carbon starvation in the induction of enzymes that degrade plant-derived carbohydrates in Aspergillus niger
Source: Fungal Genet Biol. 2014 Nov;72:34–47. doi: 10.1016/j.fgb.2014.04.006 (PMC4217149; doi:10.1016/j.fgb.2014.04.006)
Supplement: Supplementary data 7 — Supplementary Table S7. CAZymes identified by proteome analysis. [file mmc7.docx]

**Table S7.** CAZymes identified by proteome analysis.

| Accession | Name | Annotation |
| --- | --- | --- |
| **P69328** | GlaA | glucoamylase |
| P56526 | AgdA | α-glucosidase |
| A5AAG2 | AbnC | putative endo-α-1,5-arabinanase |
| A2RAR6 | ExgA | putative exo-β-1,3-glucanase |
| A2QH21 | BgtB | β-1,3-glucanotransferase, generates β-1,6-branches |
| A2QL72 | AglA | α-galactosidase |
| A2QEJ9 | AglB | α-galactosidase |
| A2QAI7 | CbhB | cellobiohydrolase |
