# Supplementary material for: The role of carbon starvation in the induction of enzymes that degrade plant-derived carbohydrates in Aspergillus niger
Source: Fungal Genet Biol. 2014 Nov;72:34–47. doi: 10.1016/j.fgb.2014.04.006 (PMC4217149; doi:10.1016/j.fgb.2014.04.006)
Supplement: Supplementary data 8 — Supplementary Fig. S1. Enzyme activity in filtrates of carbon starved cultures. Activities against plant-derived carbohydrates, as determined by reducing carbohydrate end measurements, of incubations with filtrate of cultures starving for 2 h (white), 4 h (grey), 6 h (hashed), and 9 h (black), given as mean ± standard deviation. [file mmc8.pptx]

## Slide 1
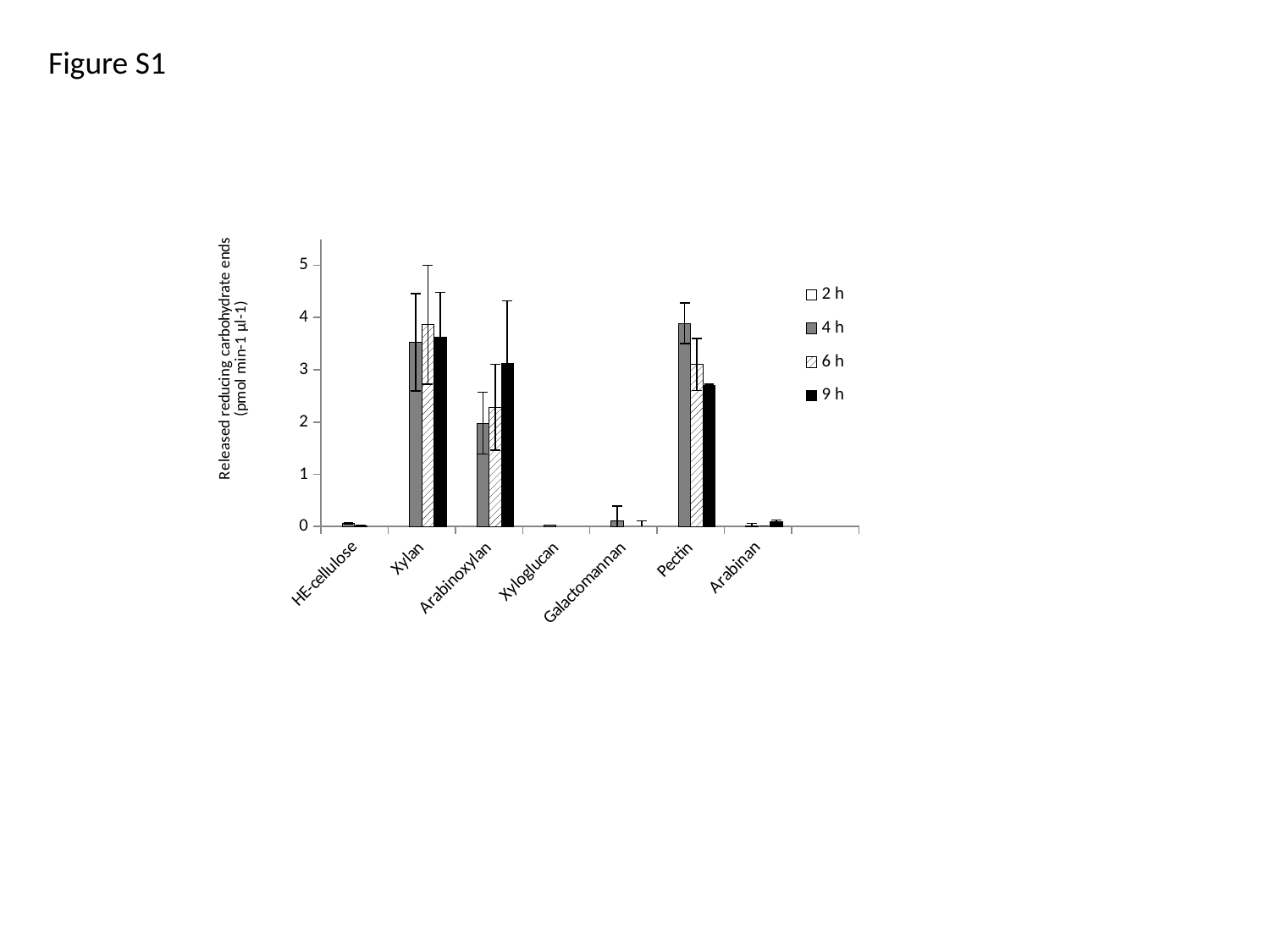

Figure S1
### Chart
| Category | | | | |
|---|---|---|---|---|
| HE-cellulose | 0.05949506685185156 | 0.06539304908620287 | 0.018312767743216184 | -0.025686008268441682 |
| Xylan | 3.0035434934999006 | 3.5264947151738815 | 3.8629095670267763 | 3.61999060795492 |
| Arabinoxylan | 1.2193239450982682 | 1.979768476884909 | 2.2803828054630833 | 3.1147296807975824 |
| Xyloglucan | -0.00791482672186618 | 0.029045836304449263 | -0.02757370199568273 | -0.029675164714554673 |
| Galactomannan | 0.034229538651216806 | 0.10772315137108393 | -0.10635593160634084 | -0.02410180621772219 |
| Pectin | 3.286408532722519 | 3.8870276946281024 | 3.103076994387637 | 2.70208286968733 |
| Arabinan | 0.05974539710603456 | 0.011688419388262181 | -0.014362360019037495 | 0.09340945416609672 |
