# Supplementary figures and images for: The role of carbon starvation in the induction of enzymes that degrade plant-derived carbohydrates in Aspergillus niger
Source: Fungal Genet Biol. 2014 Nov;72:34–47. doi: 10.1016/j.fgb.2014.04.006 (PMC4217149; doi:10.1016/j.fgb.2014.04.006)

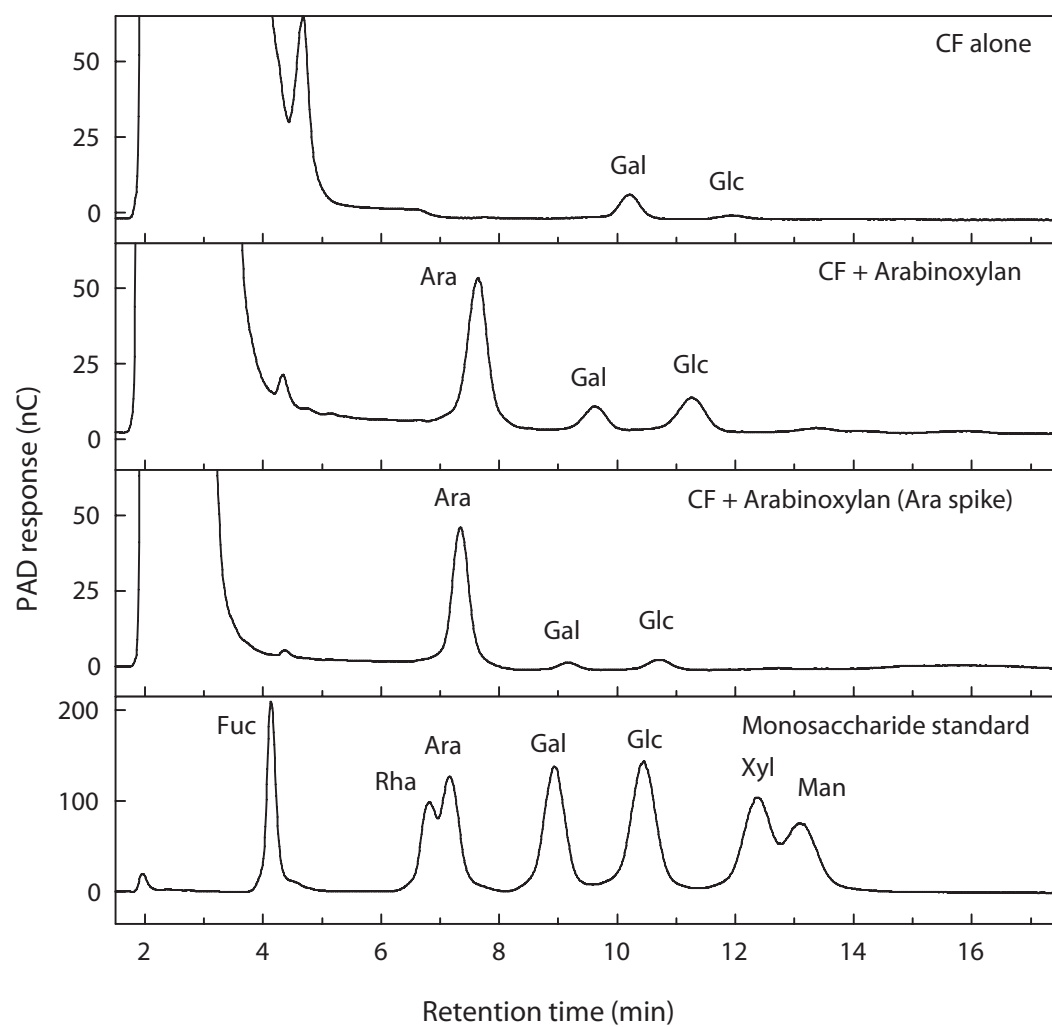

Supplement: Supplementary data 9 — Supplementary Fig. S2. HPAEC-PAD analysis of reaction products of enzyme activity against arabinoxylan in filtrates of carbon-starved cultures. The carbohydrate monomers present in the culture filtrate (A) were compared to those generated by incubation of culture filtrate with arabinoxylan (B). Identification of the reaction product as arabinose was confirmed by spiking the reaction mixture with arabinose (C). Peaks were identified by comparison to a monosaccharide standard (D) containing fucose (Fuc), rhamnose (Rha), arabinose (Ara), galactose (Gal), glucose (Glc), xylose (Xyl) and mannose (Man). [file mmc9.pdf]
